# Assessment of family function impact on depression severity among infertile women attending a teaching hospital in South-South Nigeria

**DOI:** 10.2144/fsoa-2020-0033

**Published:** 2020-06-03

**Authors:** Alex A Adelosoye, Olumuyiwa J Fasipe, Elihu I Medunoye, Onyekachukwu C Adelosoye, Elisha O Sunday

**Affiliations:** 1 Department of Family Medicine, University of Medical Sciences Teaching Hospital Complex, Ondo City, Ondo State, Nigeria; 2 Department of Clinical Pharmacology & Therapeutics, Faculty of Basic Clinical Sciences, University of Medical Sciences, Ondo City, Ondo State, Nigeria; 3 Department of Anaesthesiology, Delta State University Teaching Hospital, Oghara, Delta State, Nigeria

**Keywords:** depression, evaluation, family dysfunction, family members’ support, fertility clinics, husband support, infertility disorder, severity

## Abstract

**Aim:**

Family function and husband support can impact depression severity in women with infertility disorder. The aim of this study was to assess the impact of family function and husband support on depression severity among women with infertility disorder at the fertility clinics of a University Teaching Hospital, South-South, Nigeria.

**Methodology:**

A cross-sectional descriptive study was carried out among 341 female respondents attending the University of Benin Teaching Hospital fertility clinics over a 3-month period, using a semistructured interviewer administered questionnaire. Data obtained were analyzed.

**Result:**

Respondents mean age was 36 ± 5.3 years. The overall prevalence for depression in this study was 42.5% with a breakdown of 64.2%, 30.4% and 5.4% of these depressed participants having mild, moderate and severe depression, respectively. Family dysfunction had a statistically significant relationship with severity of depression in women with infertility (p < 0.001). A statistically significant relationship was established between poor husband support and the severity of depression (p < 0.001).

**Conclusion:**

Depression is highly prevalent among women with infertility disorder, severe depression was associated with family dysfunction. Good family function would reduce the severity of depression.

## Lay abstract

We assumed that good family function will be associated with low incidence and severity of depression in women with infertility disorder. We also reasoned that good family functioning and husband support would be very important in ameliorating depression in such women. This study assessed the incidence of depression in infertility and its severity. It investigated the association between family and husband functional relationship, vis-a-vis the severity of depression. The study revealed that depression was prevalent among women with infertility disorder at a rate of 42.5%. The majority of the depressed women had mild-to-moderate depression, with a significant association between dysfunctional family functioning and moderate-to-severe depression. Other factors such as increasing age, increase duration of infertility and polygamy affected severity of depression among women with infertility disorder in this study. Promotion of good family functioning may decrease the prevalence and severity of depression among women with infertility disorder.

Depression is a common condition associated with infertility disorder, but is poorly recognized and treated in women with infertility disorder attending many fertility clinics [1–3]. The effect of infertility on women’s mental health is an important area for research, specifically, the impact of family function or husband support on depression severity in women with infertility. Women with infertility disorder can, for diverse reasons, suffer from depressive disorder [1–3]. The social stigmatization casted upon women with infertility disorder by the African society and family members in some cultures can be very distressing [1–3]. Also, the societal expectation and demand directed at women with infertility disorder can be a source of psychological distress. In some cultures, they attribute the failure of a woman to conceive, to be woman’s problem only, while exonerating her husband [3–5]. This assumption can cause serious emotional problems between couples, and prevent them from seeking effective medical solutions. This situation can pose psychological pressure on the women much more than the men [4–6]. Worldwide, and especially in Africa, children are considered necessary ingredients of marriage and the delay or failure to have one has been associated with emotional sequelae such as anger, depression, anxiety and other forms of psychological distress [4–6]. Studies have demonstrated that depression is the most common psychological distress suffered by women with infertility disorder, with prevalence rate ranging between 40% and 50% [1–3]. Generally, women experience depression more than their male counterparts who are suffering from infertility disorder. Both men and women are affected by infertility disorder, but women tend to experience more intense psychological feelings that make them to feel anxious and depressed, because they are typically seen as the emotional caretakers of relationships [4–6]. Such feelings may affect cordiality between women and their husbands, which also include their in-laws with worsening consequences of further deterioration of family relationships. This situation usually robs women of needed support from their husbands and in-laws. These consequences can negatively affect treatment seeking behavior and adherence to fertility treatments, as well as treatment outcome [5–7].

Studies have demonstrated that couples with infertility experience a lot of psychological and emotional problems. Women tend to manifest more of these psychological problems and also experience more of the impact of these disturbances. For instance, in a study of dizygotic twins, women displayed more sensitivity to interpersonal relationships, whereas men displayed more sensitivity to external career and goal-oriented factors [6–9,28–30]. Traditional and cultural dominance of men over women in the family contribute to this problem [10,31–33]. Traditional norms in Nigeria and Africa generally accord a domineering role to the husbands [11,34,35]. A study on psychological distress among women suffering from infertility disorder in South Africa demonstrated that infertile women had significantly higher levels of distress when compared with a cohort of women attending family planning clinics for the purpose of contraception [11,12,36–38]. The husband’s family and in-laws in most cases also seek to ensure that man’s dominance is seen to be practiced and that the woman is submissive. The men determine the cultural and traditional ethos, which is often skewed in their favor as they reserve the family’s economic and decision-making power. They usually make medical assumptions and ‘diagnoses’ without consulting medical experts. As a result, women are held responsible for all cases of infertility, even though male factors have been documented to be contributory in approximately 59% of all cases in Nigeria [12–15,39–41]. A study on the direct and indirect effects of perceived social support on women’s infertility-related stress shows that social support from different sources can be related to specific coping strategies and different domains of infertility stress. The findings demonstrate that while different coping strategies can mediate the process through which social support influences infertility stress; social support from the husband and family members can directly reduce infertility-related stress in some of these domains. Healthcare professionals should explore the quality of social networks and encourage positive support from husbands, partners and family members [15–17,42,43]. Any culture where men shy away from being investigated and treated for infertility disorder, such as in Africa, is not only missing out a huge proportion of the population where intervention can be instituted, but exposes their women to unnecessary strenuous and ineffective evaluation, especially in situations where the cause of infertility is due to problems in the male [18–21]. Awareness of this situation by some women can lead to frustration and emotional trauma capable of causing depression. This can result in desperate attempt by some women trying to get pregnant through extramarital relationship [17,22]. It was found in a study on extramarital sexual activity among infertile women in South-eastern Nigeria that 38.9% of the respondents engaged in extramarital sexual relationships [17]. This situation could have far reaching and devastating effect/consequence on matrimony. On the other hand, the men easily engage in extramarital relationship and justify their actions by citing the failure of their wives to conceive as a reason. Some women are forced to accept polygamy for this reason and the men are usually supported by their relations in this regard [23].

Research conducted on the stigma of infertility in Nigeria was concluded with the finding that infertility is a major, life-altering problem in sub-Saharan Africa. It was also deduced that good community mechanisms and supportive family structures can go a long way toward mitigating its negative effects [24,25]. In the Nigerian family settings, the fact that women are held responsible for most cases of infertility disorder leave their men counterpart who may likely be significant contributor to the infertility disorder undiagnosed and untreated. This perhaps explains why women are most times the concern person who go about checking for causes and solutions to infertility in their marriages. Consequently, the women are humiliated, isolated, derided, abused and rebuffed. Undergoing such life and marital crises has been the story of most infertile women in Nigeria. They go to varying lengths, visiting orthodox medical practitioners, herbalists, spiritualists, pastors and traditionalists in search of needed reprieve and solutions to both their infertility disorder and the more devastating ensuing psychological problems [25–28]. We have seen several of such women in our clinics. Their complaints vary from vague physical pains to insomnia, nonspecific abdominal pain and crawling sensations all over the body. Some of the women may not realize that their infertility and the attendant stigmatization are responsible for this psychological distress, which is often diagnosed clinically as depression or anxiety disorder. Gynecologists who manage cases of infertility are always inundated with emerging techniques and skills of managing the ever-challenging cases of infertility where prevalence is still increasing, despite the new innovations and approaches to its management [20,25,26]. They have little or no time to attend adequately to the psychological problems of these women. It is very important for the family physicians, psychiatrists and primary care physicians to be very observant at detecting the increasing cases of depressive disorders among women with infertility disorder and attend to it appropriately, while the gynecologists focus on the fertility treatment [24–26,29,30]. This awareness would increase the use of counseling, psychotherapy and pharmacotherapy as important tools employed in the management of infertility disorder. This very essential preparation will not only reduce the incidence and severity of depression among women with infertility disorder, but will significantly improve the determination of women with infertility to follow through their management plans [25,26,31–34]. Strict adherence to treatment plans in the management of infertility disorder will encourage the healthcare providers, promote treatment outcome and reduce the disease burden on the patients, their families, the society and government. The family assessed in this study includes the husband of the woman with infertility and the extended family of the woman, especially the husband relatives (in-laws). These two entities have been identified in literature to exert huge emotional and psychological impact on these women [9,21,26–28,35].

The aim of this study was to assess the impact of family function and husband support on depression severity among women undergoing treatment for infertility disorder at the fertility clinic of University of Benin Teaching Hospital (UBTH), Benin City, Edo State Nigeria.

## Materials & methods

This study was a cross-sectional descriptive study conducted at UBTH, fertility clinics over a 3-month period between April 2013 and June 2013. The University of Benin Teaching Hospital is an 800-bed capacity teaching hospital with over 25 medical subspecialities. The fertility clinics consist of Gynecology Clinics and the Human Reproductive Research Program Unit/IVF center of the Department of Obstetrics & Gynecology. These clinics operate twice weekly on Tuesdays and Thursdays, run by a team of consultant gynecologists and fertility experts. In the same team are fertility nurses, counselors, laboratory scientists and embryologists. The services rendered included consultation, medical treatment, noninvasive and invasive investigation, minor surgical procedures and the *in vitro* fertility (IVF).

The study population was estimated to be over 10,000 adult female patients per year; that is, an average of over 200 adult females per week, aged between 20 and 45 years. The estimated sample size was calculated with the formula, N = Z^2^pq/d^2^ [22].

Where N = estimated sample size when the target population is more than 10,000,

Z = the standard deviation set at 1.96, which correspond to 95% CI,

p = the proportion of the target population estimated to be suffering from infertility using the prevalence of 43% from the Ukpong and Orji study [2],

q = 1–0.43 = 0.57 (i.e., proportion of the population free from infertility),

d = degree of accuracy desired (i.e., level of precision desired or margin of error tolerated) will be taken to be 5% = 0.05.

Therefore, N = [(1.96)^2^ × (0.43) × (0.57)]/(0.05)^2^

N = 341 participants.

By taking into account an attrition factor of 10% (which is equivalent to additional 34 participants); then, the attrition-adjusted sample size calculated equated to 375 participants.

However, only 341 respondents were found to have complete data after thorough screening and cleanup of the questionnaires that were analyzed. Nonrandom consecutive recruitment of women who met the inclusion criteria and consented to be interviewed were assessed in the study, since it was a prospective observational study carried out over a 3-month period of sample collection at the clinics with average of 50 patients per week. We used the nonrandom consecutive method approach to select respondents in order to exclude new patients attending the fertility clinics; whose medical histories put them among the ineligible individuals, and to excuse those patients who did not sign their informed consent to participate in this study.

The inclusion criteria for this study enrolled women aged 20–45 years who were unable to get pregnant for ≥2 years assuming twenty years of age as the minimum age for marriage and beginning of trial to conceive. The exclusion criteria for this study excluded: women aged 20–45 years who were unable to get pregnant for <2 years (as 2 years being the minimum duration for inability to conceive accepted by the WHO for research purposes), eligible infertile women who did not consent or failed to sign their informed consent forms, those with co-morbid chronic medical or surgical conditions capable of causing depressive disorders such as hypertension, diabetes mellitus, HIV infections, pancreatitis, cancers, autoimmune disorders and drug abuse, as well as those who had cognitive impairments, previous medical history of moderate or severe head injury, depressive disorders, substance abuse disorders or any other known psychiatric disorders.

A pretest was performed in a different setting before the main study commenced; this was to adapt the instruments and questionnaire used to improve reliability. The reliability of the measures used in this study has been adapted and validated in Nigerian clinical setting by other previous studies [2,3,6,20]. The questionnaire had four sections. Section A was on sociodemographic variables, Section B was the summary of past medical and gynecological history and Section C is the Zung’s self-rating depression assessment scale, to assess and grade severity of depression. The Zung’s questionnaire sample has a total number of 20 questions with the scoring for each question ranging from 1 to 4. The total minimum score achievable for all answered questions is 20, while the total maximum score achievable for all answered questions is 80. Normal is 20–44, mild is 45–59, moderate is 60–69 and severe depression is 70–80. Section D was the family Adaptation, Partnership, Growth, Affection and Resolve (APGAR) scoring system, which on its own is a 5-item questions used to assess for family functioning in the women examined. The family APGAR is a tool has five questions proposed to be used by physicians to assess family function. Each question is scored 0, 1 or 2 based on perception of ease or otherwise of functioning. Total score of 8–10 represent highly functional family, 4–7 is moderately dysfunctional and 0–3 severely dysfunctional family. Section E also utilizes the family APGAR questions to assess the functionality of relationship of the respondents with their husbands and other extended family members, which serves as an index of support to the women by their husbands and other extended family members, because of the critical and important role of the husbands and other extended family members in psychosocial support for women with infertility disorder. All the obtained data were analyzed using the statistical package of social sciences (SPSS) version 21.0 and STATCALC statistical software.

Results were presented using tables and figures and were expressed in mean for parametric data and percentages for nonparametric data. The Chi-square and Fisher’s exact tests were used to test for association between categorical variables. The odd ratio (OR) and 95% CI were obtained for logistic regression analysis concerning the predictors of depression among these participants. Statistical significance was set at p < 0.05. Any OR whose 95% CI reference range was not inclusive of 1.00 was considered statistically significant.

### Ethical consideration & participants’ informed consent

Ethical approval for this study was obtained from the University of Benin Teaching Hospital Ethical Research Committee. A written informed consent was also obtained, and duly signed by each of these participants recruited for this study with the assurance that confidentiality of personal information was guaranteed. The respondents who were found to be at high risk of depressive disorders were further evaluated and managed appropriately.

## Results

The modal age group of respondents was 35–39 years. The mean age was found to be 36 ± 5.3 years. Majority of the respondents, 339 (99.4%), were married and were of monogamous marriage (89.4%). More respondents had tertiary level of education (56.6%) and were employed (77.1%). In addition, 97.1% of participants were Christians. The most frequent ethnic groups were Bini (32.3%), Yoruba (19.9%), Igbo (15.8%) and Esan (13.2%) (Table 1).

**Table 1.** Demographic characteristics of respondents.

Variables	Frequency (F)	Percent (%)
**Age range**		
20–24	6	1.8
25–29	22	6.5
30–34	87	25.5
35–39	114	33.4
≥40	112	32.8
**Marital status**		
Single	2	0.6
Married	339	99.4
**Type of marriage**		
Monogamous	305	89.4
Polygamous	36	10.6
**Level of education**		
None	2	0.6
Primary	42	12.3
Secondary	104	30.5
Tertiary	193	56.6
**Employment status**		
Not employed	78	22.9
Employed	263	77.1
**Religion**		
Christianity	331	97.1
Islam	10	2.9
**Ethnicity**		
Bini	110	32.3
Esan	45	13.2
Etsako	14	4.1
Igbo	54	15.8
Yoruba	26	7.6
Urhobo	68	19.9
Others	24	7.0

Out of the 341 respondents, 230 representing 67.4% were nulliparous, while 111 representing 32.6% were parous ladies. Two hundred and forty-six (72.1%) respondents had no living child. Among the respondents, 33.1% had primary infertility, while 66.9% had secondary infertility. 36.4% of the respondents reported having no abortion in the past, 63.6% of them reportedly had at least one abortion (Table 2).

**Table 2.** Gynecological characteristics of the respondents.

Variables	Subgroups	Frequency (F)	Percent (%)
Parity	Nulliparous	230	67.4
	Parous	111	32.6
Respondents’ number of living children	None	246	72.1
	One to two	95	27.9
Respondents’ infertility type	Primary	113	33.1
	Secondary	228	66.9
History of previous abortion(s)	No	124	36.4
	Yes	217	63.6

**Figure 1.**
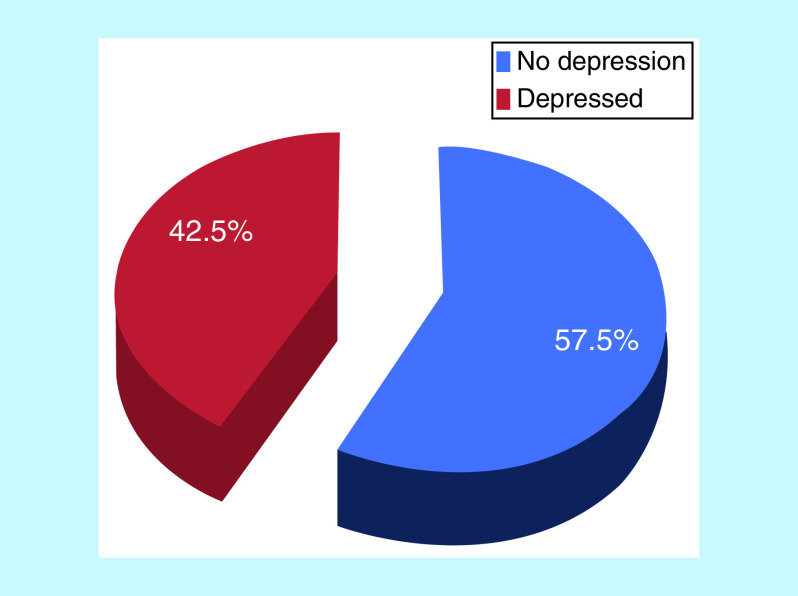
Pie chart showing the prevalence of depression among the respondents.

A total of 145 respondents met the Zung’s self-rating assessment for depression. The overall prevalence of depression among the respondents was 42.5% (Figure 1).

**Figure 2.**
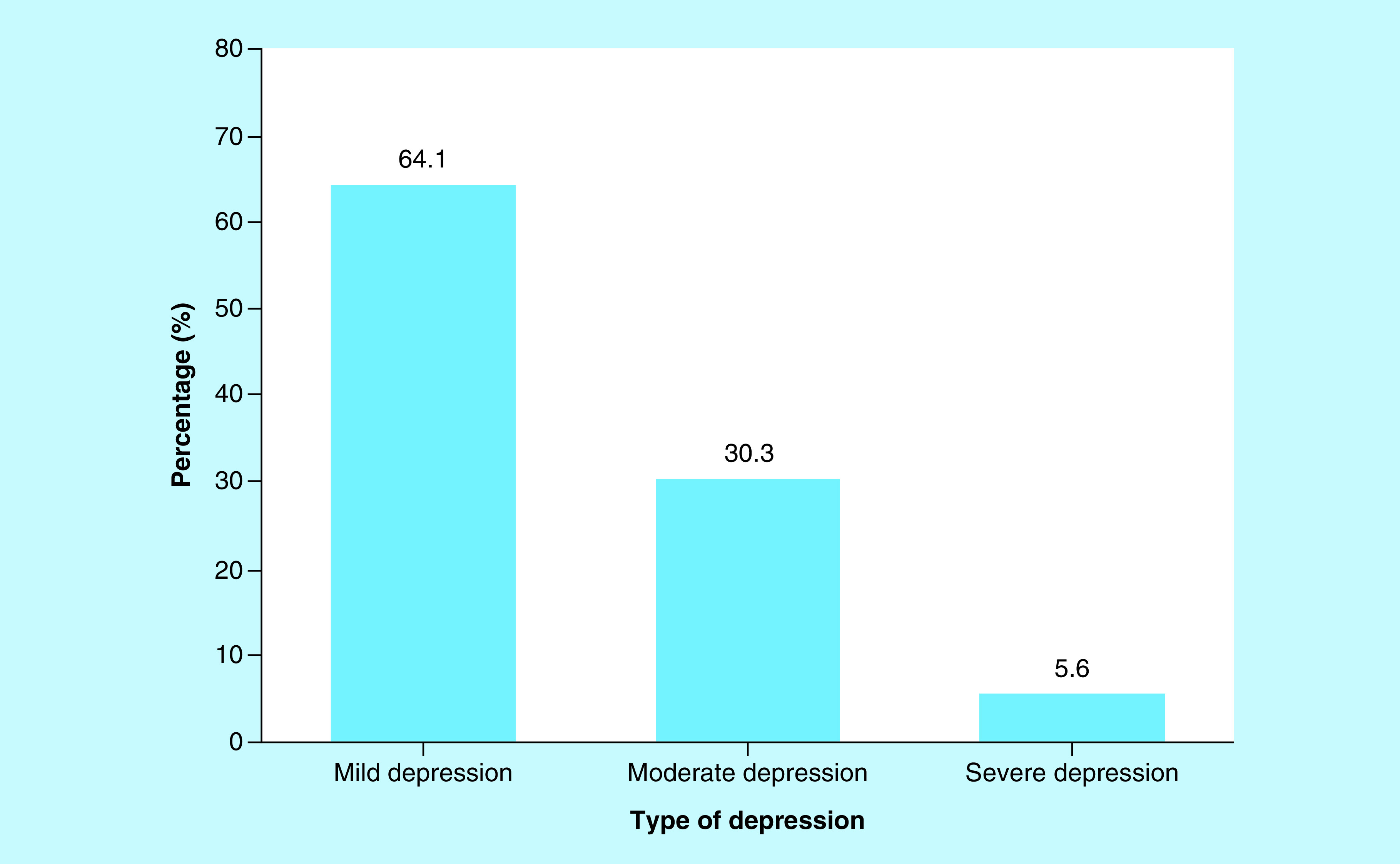
Bar chart showing severity of depression.

Of the 145 respondents with depression, 64.2% of them had mild depression, 30.4% had moderate depression and 5.4% of them had severe depression (Figure 2).

Respondents older than or equal to 35 years were more likely to be affected by depression (43.4%) when compared with those less than 35 years (40.9%); however, this association was not statistically significant. The proportion of respondents in polygamous families with depression (61.1%) was significantly more than those in monogamous families (40.3%). This association is statistically significant and the OR is 2.33 with 95% CI of 1.09–5.01. Concerning family functioning, 38% of respondents with good family function were depressed, while approximately 49% of respondents with a dysfunctional family were depressed, which was statistically significant (p = 0.044), with an OR of 0.64 (95% CI of 0.40–1.01). For husband functional support relationship, 38% of those with good husband support in the relationship had depression, while 52.3% of those with dysfunctional husband support relationship were depressed with a statistically significant value of p = 0.013 and the OR was 0.56 (95% CI of 0.34–0.91) (see Table 3).

Furthermore, there was a statistically significant association between severity of depression and family function (p < 0.001). Also, there was a statistically significant association between severity of depression and husband support (p < 0.001). This revealed that severity of depression was aggravated by dysfunctional family support and dysfunctional husband support (Table 4).

**Table 3.** Risk factors for depression among respondents.

Characteristic	Depression		p-value	OR (95% CI)
	Present n (%)	Absent n (%)		
Age group (years) – Less than 35 – 35 and above	47 (40.9) 88 (43.4)	68 (59.1) 128 (56.6)	0.659	1.11 (0.68–1.79)
Type of family – Monogamous – Polygamous	123 (40.3) 22 (61.1)	182 (59.7) 14 (38.9)	^†^0.017	2.33 (1.09–5.01)
Family functioning – Dysfunctional – Good functioning	69 (48.9) 76 (38.0)	72 (51.1) 124 (62.0)	^†^0.044	0.64 (0.40–1.01)
Husband support – Dysfunctional – Good functioning	56 (52.3) 89 (38.0)	51 (47.7) 145 (62.0)	^†^0.013	0.56 (0.34–0.91)

† Statistically significant.

OR: Odd ratio.

**Table 4.** Factors influencing the severity of depression among respondents.

Characteristic	Severity of depression			p-value
	Mild n (%)	Moderate n (%)	Severe n (%)	
Age group (years) – <35 – 35 and above	28 (59.6) 65 (66.3)	14 (29.8) 30 (30.6)	5 (10.6) 3 (03.1)	0.170
Type of family – Monogamous –Polygamous	81 (65.8) 12 (54.5)	36 (29.5) 8 (36.4)	6 (4.8) 2 (9.1)	0.526
Family functioning – Dysfunctional – Good functioning	33 (47.8) 60 (78.9)	30 (43.5) 14 (18.4)	6 (8.6) 2 (2.6)	^†^<0.001
Husband support – Dysfunctional – Good functioning	20 (35.7) 73 (82.0)	30 (53.6) 14 (15.7)	6 (10.7) 2 (02.2)	^†^<0.001

† Statistically significant.

## Discussion

This study assesses for depressive disorders among women with infertility disorder with the aim/objective of determining its prevalence, severity and how it is being influenced by family function and husband support. The modal age group in this study had 33.4% of all the respondents within the age group 35–39 years. The mean age was 36.9 ± 5.3 years. Approximately 66.2% of the entire respondents were ≥35 years of age, while 33.8% were less than 35 years. Only 8.3% of the respondents were less than 30 years. This was similar to the finding by Omoaregba *et al.* [3] in their work on women attending a fertility clinic in southern Nigeria where they found a mean age of 35.8 ± 5.9 years. Another study on female infertility in some tertiary health institutions in Nigeria revealed that the mean age of infertile women was significantly higher than those in the fertile control group [7,16]. This observation is perhaps because women who ordinarily married at an early age may not present early for fertility management, as they tend to spend time expecting to be pregnant and to overcoming inhibition forces before coming forward for treatment later. In Ukpong and Orji’s [2] work on infertile women in a teaching hospital in Ile-Ife, Nigeria, they reported a mean age of 34.5 ± 5.5 years. However, the mean age for this study contrasted with the mean age of 28.9 ± 4.1 years reported in the study by Omu and Omu [23] on infertile women in Kuwait. The lower mean age in the Omu and Omu’s [23] study reflected Kuwait culture, where majority of the women are Muslims, who due to local culture marry at an early age. Only 0.6% of the respondents were single ladies, while the remaining 99.4% were married women. Unlike the practice in the fertility clinics, where married people were primarily seen, the few single ladies that participated in this study may have come from the IVF clinic. In a similar study, the researchers highlighted that 95% of the infertile women were married and the remaining 5% were either single, separated or cohabiting [3]. Analysis of the type of marriage suggested that 89.4% of the respondents were in monogamous setting, while only 10.6% were of polygamous setting. This observation compared closely with a previous study among infertile women in Nigeria, where the respondents in monogamous and polygamous family type were 85.5 and 14.5%, respectively [2]. This distribution, where majority of the participants were in monogamous family, was perhaps because of the study location. The majority of the respondents were Christians and the majority of the participants had a higher level of educational status.

The religious distribution of the respondents in this study highlighted that approximately 97% of the respondents were Christians and approximately 3% were Muslims. This compared favorably with an earlier study performed in this environment, where a distribution of 91% Christianity, 5% Islam and 4% African traditional religion was found [3]. This could be attributed to the fact that majority of the population in southern part of Nigeria are predominantly Christians and are actively practicing.

Other findings from this study include that 56.6% of the respondents had a tertiary level of education. Other levels of education were reported as 30.5, 12.3 and 0.6% for secondary, primary and no formal education, respectively, which compared with previous studies in Ile-Ife Nigeria, where 82.1% had a tertiary level of education [2], as well as another study from Southern Nigeria where 61, 30 and 9% of the respondents had tertiary, secondary and primary level of education, respectively [6]. All of these studies were carried out in tertiary health institutions, where individuals with higher educational status tend to seek medical attention. Higher educational level and other coping factors for depression in women with infertility disorder may substantially affect the prevalence of depression among this study population, when compared with a community-based study.

Approximately 77% of our respondents were employed, while 23% were unemployed. This agrees with the study on infertility in a tertiary health institution in Nigeria [20], where they concluded that women in the infertile group were significantly more likely to be employed. Ukpong and Orji [2] determined that 87.5% of infertile women in their study were employed, while only 12.5% were not. Again, the women in the infertile group were more likely to be free to pursue employment opportunities and career progression than their fertile counterpart, who may be preoccupied with the stress of childbearing and care of their children. The employment of infertile women may also help them to cope better with psychological problems associated with infertility disorder.

Furthermore on this study, the respondents with secondary infertility were 66.9%, while those with primary infertility were 33.1%. This was in contrast to the Kuwait study on infertility where primary infertility occurred in 65.7% of the women, while 34.3% had secondary infertility [23]. The reason for this observed discrepancy may be due to socioeconomic factors and transcultural differences.

The overall prevalence of depression in this study was 42.5%. This, like findings from other previous studies, has highlighted and pin-pointed that depression is a common medical condition among women with infertility disorder. This prevalence is comparable with the findings from Ile-Ife, south-western, Nigeria, where Ukpong and Orji [2] determined a prevalence of 42.9% of depression among women with infertility disorder utilizing Beck Depression Index [2]. In that same study, the overall psychological distress was 46%. Another study in the same institution [15] determined a prevalence of 40.4% for depression in women with infertility disorder. In a study performed in Tehran, Iran, utilizing a survey to determine the relationship between anxiety, depression and duration of infertility, the investigators also noted that the prevalence of depression among the infertile women was 40.8% [4]. Conversely, a study of depression among women with infertility in Saudi Arabia reported a prevalence of 53.8% among the infertile women. This suggests that Saudi Arabian infertile women demonstrate higher rates of depression, perhaps because in Islamic countries like Saudi Arabia, family status – especially childbearing – is particularly important and these women are more likely to have polygamous family system [24]. A similar study carried out by Omoaregba *et al.* [20] in Benin City, Nigeria, assessed the psychological distress in women with infertility and highlighted that the prevalence of psychological distress was 69%. A number of studies abroad have similarly found that the occurrence and prevalence of depression in infertile women presenting for infertility treatment is significantly higher than in fertile controls [8,20,35–37]. This demonstrated that depression is a very common problem among women with infertility disorder worldwide; and this transcends racially, culturally and socioeconomically. With the prevalence recorded in this study, it can be deduced that at least two out of every five infertile women presenting for fertility treatment had clinical features of depressive disorders. Clinical depression is classified as mild, moderate and severe depression [11,38–40]. Findings from this study showed that 57.5% of the respondents did not show signs and symptoms satisfying the criteria for depression.

Furthermore in this study, out of the 42.5% infertile women that had depression; 64.2%, 30.4% and 5.4% of them had mild, moderate and severe depression, respectively. This is similar to the finding by Bell *et al.* [13] who determined a severity rate of 77.1%, 19.6% and 3.3% for mild, moderate and severe depression, respectively. In addition, a study by Ramezanzadeh *et al.* [4] among infertile women reported that 63.5% had mild depression, 24.5% had moderate depression and 12% had severe depression. This may be due to the nature of the Iranian society where infertility disorder bears more emotional impact because of the religion of Islam and tendency to be to polygamous as a result of infertility disorder. This study has shown that majority of the participants (94.6%) have mild-to-moderate depression and so the use of psychotherapy with or without pharmacotherapy will address this problem in most of them.

Using the family APGAR as an index of family function assessment, there was a statistically significant association between family function and severity of depression (p < 0.001). More of those with good family function presented with no signs of depression, while those with family dysfunction showed signs of increased depression severity. It should be noted that lack of support from the family – especially the husband’s family, which is the case in many infertile women in Africa – is a strong predictor of depression severity and other psychological problems associated with infertility disorder [12,14,42,43]. The importance of support from the husband was also assessed in this study using the family APGAR, to check for women’s satisfaction concerning the support and the functionality of the relationship they enjoyed with their husbands. This was also measured against the severity of depression and it was discovered that there was a statistically significant strong association between husbands support and severity of depression (p < 0.001). Concerning family functioning, 38% of the respondents with good family function are depressed, whereas approximately 49% of the respondents with a dysfunctional family are depressed, and this was statistically significant. For husband functional support relationship, 38% of those with good functional husband support have depression, while 52.3% of those with dysfunctional husband support are depressed. This is statistically significant and the OR that an infertile woman with good-functioning husband support relationship will have depression is approximately half (0.56) of the value for an infertile woman with dysfunctional husband support. This agreed with the findings from other previous studies performed [2,3,15,21,23,44–46], confirming that good husband support plays a vital role in predicting depression severity and psychological distress in women with infertility disorder, as lack of positive support from the husbands will predict poor psychiatric outcome.

The sociodemographic characteristics of the respondents studied in comparison to the severity of depression in this study included age, types of family and types of infertility disorder. Respondents older than or equal to 35 years recorded reported a higher prevalence of depression (43.4%) than those less than 35 years (40.9%); however, this association was not statistically significant. Regarding the type of family setting in comparison to the occurrence of depression, as expected, the proportion of respondents in polygamous family with depression (61.1%) was significantly more than those in monogamous family (40.3%). This association was statistically significant (p = 0.017) with an OR at of 2.33 (95% CI: 1.09–5.01). This implies that the OR for a depressed woman in polygamous family setting with infertility disorder is more than twice of her depressed counterpart in monogamous family setting. The other risk factors for the occurrence of depression among these infertile women involved in this study were parity, presence or absence of living offspring in the couples’ matrimony and duration of infertility disorder. It was observed that with increasing age, there was gradual increase in the proportion of respondents with depression and the degree of depression severity compared with those without depression. Duration of infertility in this study was also found to predict the occurrence of depression in women with infertility disorder. While the majority of the respondents living with infertility disorder for less than 10 years were not depressed, the majority of those with infertility disorder ≥10 years were depressed. This was statistically significant with p-value of 0.004.

The limitation of this study was that it enrolled only consented infertile women who were gynecological outpatients being managed for infertility disorder. Also, this study could not for a very important reason of excluding ineligible patients employed a systematic random sampling technique method in the process of recruiting respondents.

## Conclusion

The prevalence of depression among women with infertility disorder in this study is significant. About two-fifths of women treated for infertility disorder had depression. The majority of the depressed had mild-to-moderate depression (94.5%). This large proportion of women can be treated at a primary care level using counseling/psychotherapy, with or without pharmacotherapy. There is a statistically significant association between family functioning, husband support and depression severity. The OR for a depressed woman in polygamous family setting with infertility disorder is more than twice of her depressed counterpart in monogamous family setting. The other risk factors for the occurrence of depression among these infertile women involved in this study included, parity, presence or absence of living offspring(s) in the couples’ matrimony and duration of infertility disorder. It was observed that with increasing age, there was gradual increase in the proportion of respondents with depression and the degree of depression severity compared with those without depression. Promotion of good family function through proper emphasis on husband and family members support, with a view to promote harmonious relationship, can reduce the prevalence and severity of depression among women with infertility disorder.

## Future perspective

Future research is required to identify if there is a similar occurrence of depression and infertility among men and to compare male and female perceptions of the burden of infertility.

Other associated factors such as family income and well-being can also be studied in relation to psychological distress in infertility. Other factors that can improve the psychological distress of the women can also be looked into, including the role of adoption and its acceptance in infertility management.

The assessment of results of improved family functioning and early detection and treatment of mild-to-moderate depression among couples with infertility should studied.

**Summary points**

- We postulated that depression is highly prevalent among women with infertility. We also believed that poor family functioning and lack of good support from husbands of such women could be associated with increased incidence of depression and increased severity.
- This study assessed the prevalence, severity of depression and relationship between family function, husband support and depression.
- The modal age group constituting 33.4% of all the respondents was 35–39 years. The mean age was 36.9 ± 5.3 years. Approximately two-thirds of the respondents were ≥35 years of age. 89.4% of the respondents were in monogamous setting and 10.6% were of polygamous setting. The majority of the respondents, 97% were Christians and 3% were Muslims.
- 56.6% of the respondents had tertiary level of education. 30.5, 12.3 and 0.6% had secondary, primary and no formal education, respectively. 77% of our respondents were employed while approximately 23% were unemployed. Respondents with secondary infertility accounted for 66.9%, while 33.1% had primary infertility.
- The overall prevalence of depression from this study was 42.5%. Of these, 64.2, 30.4 and 5.4% of them had mild, moderate and severe depression, respectively. This study has demonstrated that majority of the participants (94.6%) have mild-to-moderate depression and so the use of psychotherapy with or without pharmacotherapy could address this problem.
- There was a statistically significant association between family function and severity of depression (p < 0.001). More of those with good family function showed no signs of depression, while more of those with family dysfunction showed signs of increased depression severity. Lack of good family functioning especially the husband’s family is a strong predictor of depression severity. There was also a statistically significant relationship between husbands support and severity of depression (p < 0.001).
- The chance that a woman with infertility disorder will be depressed is twice for those in polygamous family setting and dysfunctional relationship with their husband, when compare with those in monogamous family and good relationship with their husbands, respectively.
- Respondents ≥35 years were more affected by depression than those less than 35 years. Those with higher level of education were less likely to develop depression compared with those with low level of education. Only 40.3% of respondents in monogamous marriage were depressed, while 61.1% of those in polygamous marriage were depressed, and this was a statistically significant association (p = 0.017).
- The promotion of good family function, through proper recruitment of husband and family members support, may reduce the prevalence and severity of depression in women with infertility disorder.
